# Predicting the risk of postoperative constipation in middle-aged and elderly patients with lower limb fractures using machine learning algorithms

**DOI:** 10.1371/journal.pone.0336466

**Published:** 2025-11-21

**Authors:** Xiaoyan Yang, Wenqiang Li, Qin Xiao, Shiyun Du, Xi Wang, Ying Zhang, Sulian Li

**Affiliations:** 1 Southwest Medical University, Luzhou, China,; 2 The Affiliated Traditional Chinese Medicine Hospital, Southwest Medical University, Luzhou, China; King Fahad Medical City, SAUDI ARABIA

## Abstract

**Objective:**

To construct and validate a predictive model for the risk of postoperative constipation in middle-aged and elderly patients with lower limb fractures based on machine learning algorithms, so as to provide decision-making support for clinical prevention and early intervention.

**Methods:**

This study conducted a retrospective analysis of clinical data of 1,128 middle-aged and elderly patients who underwent lower limb fracture surgery between January 2020 and May 2024, with data collection occurring from October to December 2024. Whether constipation occurred or not was used as the outcome variable. Eight machine learning algorithms, namely logistic regression (LR), extreme gradient boosting (XGBoost), random forest (RF), decision tree classifier (DT), complement naive bayes (CNB), multilayer perceptron (MLP), support vector machine (SVM), and K-Nearest neighbors (KNN), were employed to construct predictive models. Key risk factors were identified using SHAP (SHapley Additive exPlanations), a game theory-based approach for analyzing feature importance. Model predictive performance was comprehensively evaluated using metrics including the area under the receiver operating characteristic curve (AUC), accuracy, and other relevant indicators.

**Results:**

The logistic regression (LR) model demonstrated the optimal predictive performance. Age, femoral fracture, length of hospital stay, nutritional risk, and chronic gastritis were identified as important predictive factors. This model can be integrated into the clinical information system to automatically flag high-risk patients upon admission and provide individualized interventions based on risk stratification.

**Conclusion:**

The logistic regression (LR) model developed in this study exhibits strong discriminative ability and clinical utility, enabling dynamic perioperative monitoring of constipation risk through digital health tools, thereby potentially reducing related complications.

## Introduction

With the accelerating global aging process, fractures caused by bone fragility have emerged as a significant public health concern. It is projected that the global annual incidence of fractures will reach 5.91 million cases by 2050 [1,2]. In addressing this public health challenge, the management of postoperative complications has become a pivotal factor influencing clinical outcomes. Current evidence indicates that 71.7% of femoral neck fracture patients and 50% of orthopedic surgery patients develop postoperative constipation [3]. This complication not only lead to gastrointestinal discomfort symptoms such as abdominal distension, loss of appetite, and nausea but may also induce severe secondary conditions ranging from electrolyte imbalances to cardiovascular and cerebrovascular events [4]. Consequently, it directly results in prolonged hospital stays, increased medical costs, and exacerbates the socioeconomic burden. Therefore, particular attention should be paid to postoperative constipation in middle-aged and elderly patients with lower extremity fractures. Although existing studies have identified scattered risk factors such as age and postoperative bed-rest time [5], there is a lack of systematic prediction tools. Machine learning technology is triggering a paradigm shift in the medical field. Compared to traditional statistical models, artificial intelligence (AI) models possess unique advantages in handling high-dimensional variables and the complex interactions and non-linear relationships among variables. Their dynamic learning mechanisms enable continuous optimization of predictive accuracy as sample sizes increase. Furthermore, advancements in interpretability techniques (e.g., SHAP [SHapley Additive exPlanations]) have significantly mitigated the “black box” problem [6,7]. Multiple model algorithms, including Logistic regression, random forest (RF), and support vector machine (SVM), have been widely applied in disease diagnosis and risk model prediction [8–10]. In the orthopedic field, previous studies have successfully employed random forest to predict deep vein thrombosis [11]. However, there remains a significant research gap in developing prediction models for postoperative constipation-a highly prevalent complication among middle-aged and elderly patients with lower extremity fractures. In this study, by screening multiple variable factors, the variables with differences were used to construct a predictive model for the risk of postoperative constipation in middle-aged and elderly patients with lower limb fractures using machine learning algorithms and systematically comparing the predictive performance of eight distinct algorithms. Our approach provides quantifiable decision support for Enhanced Recovery After Surgery (ERAS) protocols, enabling more precise preventive interventions through a predictive-to-interventional closed-loop management system. This advancement holds significant potential for improving patient quality of care.

## Methods and participants

### Study participants

**Fig 1:**
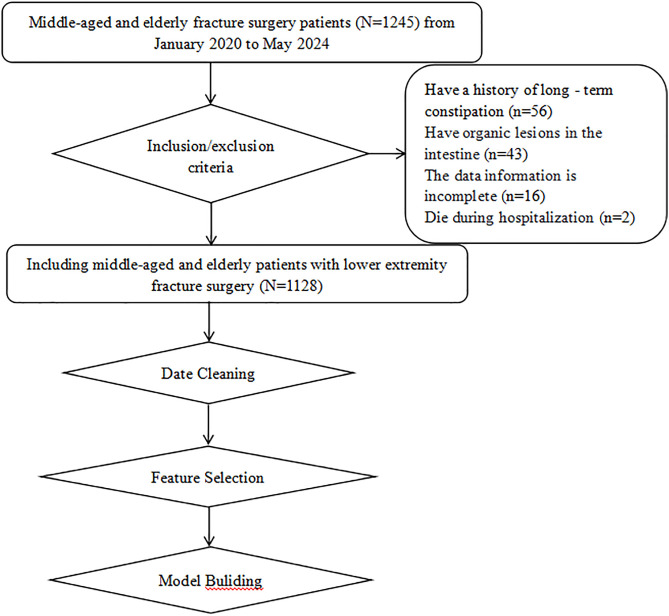
The patient selection procedure.

Using convenience sampling, we collected clinical data from 1,128 middle-aged and elderly patients who underwent fracture surgery between January 2020 and May 2024 at a tertiary Grade A hospital in Sichuan Province from October to December 2024. Inclusion criteria: (1) Age ≥ 45 years old; (2) Meeting the diagnostic criteria for lower limb fractures; (3) Patients who underwent surgical treatment after admission. Exclusion criteria: (1) Patients with a history of long-term constipation; (2) Patients with organic intestinal diseases; (3) Patients with incomplete data information; (4) Patients who died during hospitalization. The patient flow screening is shown in Fig 1. This study was approved by the Ethics Committee of Affiliated Hospital of Traditional Chinese Medicine, Southwest Medical University (approval number: BY2024031). As this study is retrospective, patients are exempt from providing informed consent.

### Methods

#### Data collection.

The following data were collected through the hospital’s electronic medical record information system:

(1) Socio-demographic data, including age, gender, body mass index (BMI), occupation, smoking history, and alcohol consumption history.
(2) Medical history data: osteoporosis, hypertension, diabetes, coronary heart disease, cerebral infarction, length of hospital stay, fracture site (classified according to postoperative X-ray reports as femoral neck/intertrochanteric/tibiofibular etc.), chronic gastritis (confirmed by gastroscopy reports) and nutritional risk (assessed using the NRS 2002 scale for malnutrition and diagnosed with hypoproteinemia based on blood biochemical indicators).
(3) Outcome indicators: Constipation was diagnosed according to the Rome IV criteria [12], and the relevant definition of constipation after orthopedic surgery proposed by Mantegazzi was also referred to [13,14]. Two senior nurses independently evaluated all cases to ensure diagnostic consistency:

① At least one objective symptom:
  - No bowel movement for >72 hours postoperatively;
  - Difficult defecation (straining >25% of effort);
  - Painful defecation.
② ≥ 2 subjective symptoms:
  - Reduced stool volume (Bristol Stool Scale type 1–2)
  - Abdominal pain (VAS ≥ 4);
  - Bloating/distension;
  - **Loss of appetite**

③ If the patient required enema for bowel movement, it was considered that the patient had constipation (documented after 48h of no bowel movement + surgeon approval).

#### Data preprocessing.

The data preprocessing procedure included data cleaning and feature processing. Variables with missing values exceeding 20% of the total data were removed, while those with less than 20% missing values were imputed. For continuous variables, mean imputation was employed, whereas random imputation was applied for categorical variables. Multiclass categorical variables including gender, education level, and fracture site were independently encoded. Finally, min-max normalization was performed on the dataset to ensure data consistency for subsequent analyses.

#### Statistical methods.

The data were organized and analyzed using the R statistical software. Data conforming to the normal distribution were expressed as means ± standard deviation (*x ± s*), data not conforming to the normal distribution were expressed as median, interquartile range (*IQR*), and count data were expressed as percentage (%). The t-test was used for comparison between groups. In this study, univariate analysis was conducted first. Then, variables with statistical significance (*P* < 0.05) were screened out through multivariate Logistic regression. Finally, the model was constructed using Python software.

#### Model development.

Models were constructed based on eight machine-learning algorithms, namely logistic regression (LR), extreme gradient boosting (XGBoost), random forest (RF), Decision Tree, Complement Naive Bayes (CNB), multilayer perceptron (MLP), support vector machine (SVM), and K-Nearest Neighbors (KNN). The hyperparameters for each algorithm are presented in Table 1. First, the dataset was randomly divided into a test set and a training set at a ratio of 8:2. During the training process, the 10-fold cross-validation method was adopted. Each model was evaluated by the receiver operating characteristic curve (ROC curve) and the area under the curve (AUC), sensitivity, specificity, accuracy, and F1-score [15,16]. Shapley Additive Explanation (SHAP) was applied to explain the prediction model with the best performance, and the relevant SHAP values were calculated [17]. Both SHAP value plots and variable importance plots were used to display the contribution of each feature to the model and their respective importance rankings.

**Table 1:** Parameters of the machine learning algorithms used in the study.

Algorithms	Parameters
LR	C = 1.0
	Maximum Number of Iterations = 100
	Penalty: l2
	Tolerance for Stopping Criteria = 0.0001
XGBoost	Objective: binary: logistic
	Learning_rate: None
	Maximum Tree Depth: None
	Minimum Sum of Instance Weight: None
	Reg_lambda: None
RF	Criterion: gini
	Maximum Tree Depth: None
	Minimum Impurity Reduction = 0.0
	N_estimators = 20
DT	Criterion: gini
	Maximum Tree Depth: None
	Minimum Samples per Leaf = 1
	Minimum Samples to Split = 2
CNB	Alpha = 1.0
MLP	Activation: relu
	Hidden_layer_sizes: (20, 10)
	Maximum Iterations = 20
SVM	C = 1.0
	Kernel: rbf
	Tolerance for Stopping Criteria = 0.001
KNN	N_neighbors = 5
	Weights: uniform

## Results

### Demographic data

A total of 1,128 middle-aged and elderly patients who underwent lower limb fracture surgery were included, with an age of (70 ± 10) years old. There were 719 females and 409 males. Table 2 shows the baseline data of the patients. According to whether the patients had constipation, they were divided into a constipation group (n = 386) and a non-constipation group (n = 742). Univariate analysis was carried out on the general data of the two groups of patients. There were statistically significant differences in age, body mass index (BMI), fracture site, osteoporosis, chronic gastritis, nutritional risk, cerebral infarction, diabetes, hypertension, and coronary heart disease between the two groups of patients (*P* < 0.05). There were no statistically significant differences in gender, occupation, surgical history, smoking history, and drinking history (*P* > 0.05).

**Table 2:** Comparison of Baseline Data between the Two Groups of Patients.

Variable, n (%)	Constipation group(n = 742) %	Nonconstipation group(n = 386) %	*X* ^*2*^ */Z*	*p*
Gender			0.158	0.691
Female	476(64.15)			243(62.95)
Male	266(35.85)			143(37.04)
Occupation			6.083	0.108
Other	561(75.61)			269(69.69)
Retiree	66(8.89)			50(12.95)
Labor	11(1.48)			8(2.07)
Peasant	104(14.01)			59(15.28)
Fracture site			70.094	<0.001***
Femoral fracture	249(33.55)			229(59.32)
Knee fracture	258(34.77)			74(19.17)
Other fractures of the lower extremities	235(31.67)			83(21.50)
History of surgery			0.000	0.992
No	623(83.96)			324(83.93)
Yes	119(16.03)			62(16.06)
Osteoporosis			15.086	<0.001***
No	224(30.18)			75(19.43)
Yes	518(69.81)			311(80.57)
Chronic gastritis			6.162	0.013*
No	658(88.67)			322(83.42)
Yes	84(11.32)			64(16.58)
Nutritional risk			51.776	<0.001***
No	592(79.78)			231(59.84)
Innutrition	26(3.50)			32(8.29)
Hypoproteinemia	124(16.71)			123(31.86)
Cerebral infarction			38.679	<0.001***
No	586(78.97)			238(61.65)
Yes	156(21.02)			148(38.34)
Diabetes mellitus			10.906	<0.001***
No	649(87.46)			309(80.05)
Yes	93(12.53)			77(19.94)
Hypertension			12.904	<0.001***
No	463(62.39)			198(51.29)
Yes	279(37.60)			188(48.70)
Coronary disease			7.765	0.005**
No	681(91.77)	334(86.52)		
Yes	61(8.22)	52(13.47)		
Smoke			0.703	0.402
No	604(81.40)			322(83.42)
Yes	138(18.59)			64(16.58)
Drink			0.189	0.664
No	617(83.15)			317(82.12)
Yes	125(16.84)			69(17.87)

Note: *** Significant; ** Moderately significant; * Significant.

### Logistic multivariate regression analysis

Multivariate logistic regression analysis was performed with postoperative constipation (coded as 0 = no, 1 = yes) in middle-aged and elderly patients with lower extremity fractures as the dependent variable, and factors showing significant differences in univariate analysis as independent variables. As presented in Table 3, age, length of hospital stay, fracture site, chronic gastritis, and nutritional risk were identified as significant influencing factors for postoperative constipation (P < 0.05).

**Table 3:** Results of Multivariate Logistic Regression Analysis.

Predictor	*P*	*OR*	*95%LCI*	*95%UCI*
Length of stay	0.0	1.08	1.056	1.106
Age	0.0	1.056	1.041	1.072
^*^Fracture Site 2	0.001	0.539	0.369	0.785
^*^Fracture Site 3	0.018	0.651	0.456	0.927
Chronic gastritis	0.144	1.347	0.901	2.007
^*^Nutrition Risk 1	0.003	2.508	1.365	4.638
^*^Nutrition Risk 2	0.0	1.936	1.4	2.677

Abbreviations: OR, odds ratio; LCI, lower confidence interval; UCI, upper confidence interval.

*Fracture site 2 (Knee fracture)/3 (Other fractures of the lower extremities); nutritional risk 1(malnutrition)/2 (hypoproteinemia).

### Model construction and evaluation

The variables screened out, namely age, length of hospital stay, fracture site, chronic gastritis, and nutritional risk, were used to construct risk prediction models with eight machine-learning algorithms (LR, XGBoost, RF, Decision Tree, CNB, MLP, SVM, KNN). To evaluate the prediction models and prevent overfitting, we employed a 10-fold cross-validation approach for model training and validation. This method partitions the entire dataset into 8 training subsets and 2 validation subsets in each iteration, ensuring all data points are used for both training and testing. Through this process, we calculated the AUC values, accuracy, sensitivity, specificity, and F1-scores for all eight prediction models using confusion matrices (Table 4).

**Table 4:** Evaluation metrics of the models constructed by each algorithm.

Model name	*AUC(95%CI)*	*ACC(95%CI)*	*SEN(95%CI)*	*SPE(95%CI)*	*PPV(95%CI)*	*NPV(95%CI)*	*F1score(95%CI)*	*Kappa(95%CI)*
**Train**								
LR	0.743(0.733-0.753)	0.702(0.665-0.739)	0.623(0.509-0.737)	0.742(0.628-0.856)	0.561(0.510-0.612)	0.793(0.762-0.824)	0.588(0.568-0.608)	0.356(0.323-0.389)
XGBoost	0.965(0.959-0.971)	0.899(0.883-0.915)	0.899(0.856-0.942)	0.900(0.857-0.943)	0.825(0.768-0.882)	0.945(0.925-0.965)	0.860(0.844-0.876)	0.782(0.753-0.811)
RF	0.992(0.990-0.994)	0.949(0.941-0.957)	0.971(0.949-0.993)	0.938(0.918-0.958)	0.892(0.865-0.919)	0.984(0.972-0.996)	0.929(0.919-0.939)	0.890(0.874-0.906)
DT	0.996(0.994-0.998)	0.958(0.952-0.964)	0.987(0.979-0.995)	0.942(0.930-0.954)	0.899(0.881-0.917)	0.993(0.989-0.997)	0.941(0.933-0.949)	0.909(0.897-0.921)
CNB	0.654(0.642-0.666)	0.637(0.604-0.670)	0.610(0.539-0.681)	0.650(0.566-0.734)	0.477(0.444-0.510)	0.763(0.751-0.775)	0.534(0.520-0.548)	0.244(0.211-0.277)
MLP	0.647(0.623-0.671)	0.649(0.631-0.667)	0.515(0.421-0.609)	0.720(0.647-0.793)	0.489(0.466-0.512)	0.741(0.721-0.761)	0.500(0.463-0.537)	0.231()0.207-0.255
SVM	0.737(0.729-0.745)	0.674(0.662-0.686)	0.688(0.651-0.725)	0.666(0.633-0.699)	0.518(0.504-0.532)	0.804(0.792-0.816)	0.590(0.580-0.600)	0.328(0.312-0.344)
KNN	0.8380.824-0.852()	0.728(0.712-0.744)	0.847(0.823-0.871)	0.667(0.649-0.685)	0.569(0.553-0.585)	0.893(0.877-0.909)	0.681(0.663-0.699)	0.460(0.431-0.489)
**Valid**								
LR	0.736(0.658-0.814)	0.678(0.586-0.770)	0.580(0.427-0.733)	0.729(0.584-0.874)	0.533(0.398-0.668)	0.770(0.711-0.829)	0.552(0.438-0.666)	0.304(0.126-0.482)
XGBoost	0.666(0.546-0.786)	0.650(0.560-0.740)	0.541(0.374-0.708)	0.706(0.622-0.790)	0.489(0.371-0.607)	0.748(0.670-0.826)	0.513(0.380-0.646)	0.241(0.043-0.439)
RF	0.669(0.571-0.767)	0.630(0.561-0.699)	0.518(0.365-0.671)	0.689(0.593-0.785)	0.465(0.377-0.553)	0.734(0.675-0.793)	0.488(0.384-0.592)	0.201(0.056-0.346)
DT	0.567(0.461-0.673)	0.595(0.499-0.691)	0.445(0.272-0.618)	0.672(0.580-0.764)	0.413(0.288-0.538)	0.700(0.620-0.780)	0.428(0.285-0.571)	0.115(−0.095-0.325)
CNB	0.650(0.521-0.779)	0.627(0.531-0.723)	0.591(0.407-0.775)	0.646(0.536-0.756)	0.465(0.343-0.587)	0.753(0.663-0.843)	0.519(0.385-0.653)	0.221(0.017-0.425)
MLP	0.640(0.518-0.762)	0.629(0.543-0.715)	0.490(0.372-0.608)	0.701(0.603-0.799)	0.462(0.358-0.566)	0.725(0.658-0.792)	0.475(0.371-0.579)	0.188(0.016-0.360)
SVM	0.732(0.679-0.785)	0.660(0.589-0.731)	0.663(0.590-0.736)	0.658(0.572-0.744)	0.503(0.425-0.581)	0.789(0.742-0.836)	0.572(0.499-0.645)	0.299(0.170-0.428)
KNN	0.666(0.572-0.760)	0.607(0.505-0.709)	0.671(0.563-0.779)	0.574(0.462-0.686)	0.452(0.356-0.548)	0.769(0.685-0.853)	0.540(0.438-0.642)	0.220(0.038-0.402)

Train, Training set; Valid, Validation set; AUC, Area under the curve; ACC, Accuracy; SEN, Sensitivity; SPE, Specificity; PPV, Positive predictive value; NPV, Negative predictive value; CI, confidence interval.

**Fig 2:**
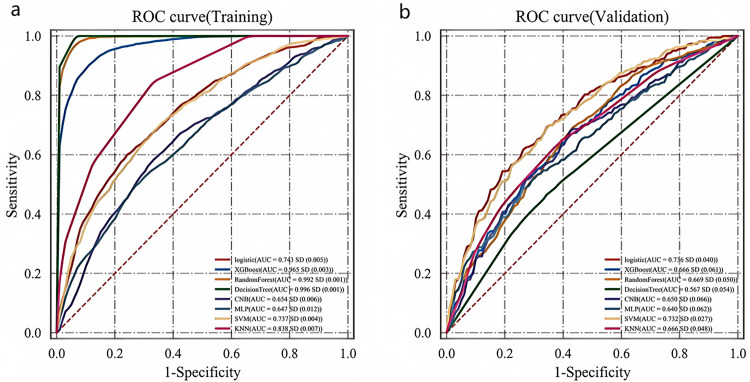
ROC curves for each model in the training and validation sets. (a) ROC curves in the training set; (b) ROC curves in the validation set.

**Fig 3:**
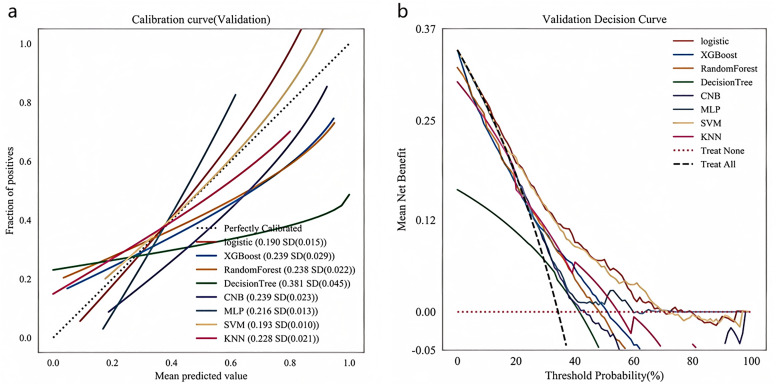
Calibration curves and DCA curves for each model in the validation set. (a) Calibration curves in the validation set; (b) DCA curves in the validation set.

The results demonstrated that in the training set, the AUC values for LR, XGBoost, RF, DT, CNB, MLP, SVM, and KNN algorithms were 0.743, 0.965, 0.992, 0.996, 0.654, 0.647, 0.737, and 0.838, respectively (Fig 2a). Corresponding AUC values in the validation set were 0.736, 0.666, 0.669, 0.567, 0.650, 0.640, 0.732, and 0.666 (Fig 2b). The logistic regression (LR) model showed the smallest performance discrepancy between training and validation sets (ΔAUC = 0.007), indicating superior generalization capability. Moreover, its other metrics (F1 = 0.552, Kappa = 0.304) significantly outperformed other models. Notably, XGBoost, RF, and DT models achieved exceptionally high training AUCs (>0.96) but exhibited substantial performance degradation in validation (ΔAUC>0.25), with DT performing particularly poorly (AUC = 0.567), suggesting overfitting. Comprehensive evaluation incorporating calibration curves (Fig 3a) and decision curve analysis (Fig 3b) from the validation set confirmed that the LR-based model delivered optimal and most stable performance, followed by SVM, XGBoost and KNN algorithms Fig 3.

**Fig 4:**
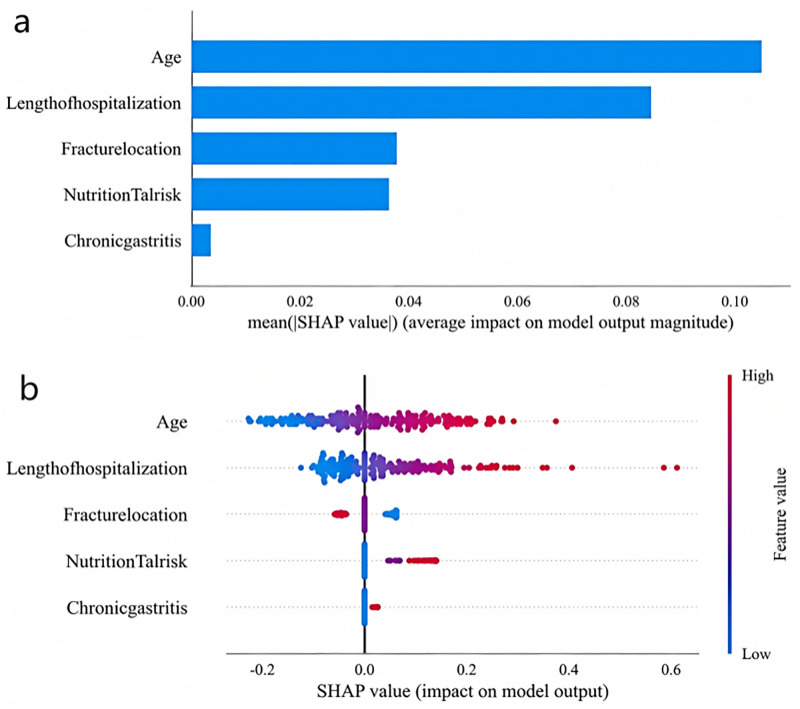
SHAP values and feature interaction scores in logistic regression (LR)-based prediction. (a) Contributing factors were ranked in descending order of importance; (b) The distribution of the impacts of each feature on the model output.

We used the logistic regression (LR) machine-learning algorithm to build a prediction model, and further calculated the SHAP value of each feature to understand the contribution of features in the prediction model based on the logistic regression (LR) algorithm to the prediction results. Fig 4a shows the importance ranking of each feature according to the mean absolute SHAP value. The larger the absolute value of the SHAP of a feature, the more it implies that the feature has a greater impact on the LR-based prediction model. The results show that four features, namely age, length of hospital stay, fracture site, and nutritional risk, are important variables affecting the outcome. Fig 4b shows the correlation between the value of each feature and the SHAP value. Red dots represent higher values of this feature, while blue dots represent lower values of this feature.

## Discussion

Constipation, as a prevalent complication, frequently occurs during the postoperative recovery period of fractures in multiple anatomical sites, including the hip and lower limbs. It is mainly characterized by insufficient defecation or difficult bowel movements, accompanied by abdominal pain and distension [18,19]. The onset of constipation can exert a profound impact on patients [20,21]. Firstly, constipation can impair the gastrointestinal barrier function of patients, elevating the incidence of other gastrointestinal disorders [22]. In extreme cases, it may even trigger cardiovascular and cerebrovascular diseases during forced defecation. Secondly, constipation can further deteriorate the nutritional status of patients, leading to psychological issues such as irritability and anxiety [23]. In severe instances, it may impede fracture healing, prolong the length of hospital stay, and diminish the patients’ quality of life [24,25]. Therefore, systematically predicting the risk of postoperative constipation in middle-aged and elderly patients with lower limb fractures facilitates early detection and timely intervention, thereby reducing the incidence of secondary complications. This is of great significance for improving patients’ prognosis.

In recent years, with the continuous increase in the quantity and complexity of biomedical data, machine-learning (ML) methods are becoming popular tools for creating predictive models in biomedicine [26]. Disease prediction models can combine two or more pieces of relevant patient data to predict clinical outcomes, assist in medical decision-making, and improve clinical work efficiency. In this study, eight machine-learning algorithms were employed to construct models for predicting the risk of constipation after lower limb fracture surgery in middle-aged and elderly patients based on their clinical data. We found that the prediction model based on the LR algorithm showed the best performance. This model’s predicted probabilities were closer to the actual situation, indicating excellent clinical utility [27]. This observation may be attributed to the predominance of binary variables in our dataset and the sample size aligning with LR’s superior performance characteristics for “small-sample, low-dimensional” data. Compared to previous studies developing and validating nomograms for predicting postoperative constipation in elderly patients undergoing hip fracture surgery [14], our machine learning approach not only identified the optimal predictive model through comprehensive algorithm comparison, but also demonstrated higher AUC values in both training and validation sets using the LR model.

Our study employed SHAP analysis to interpret the predictive model, revealing that age, length of hospital stay, fracture site, nutritional risk, and chronic gastritis were significant risk factors for postoperative constipation after fractures. These findings are partially supported by previous research. Notably, several studies have identified constipation as one of the most common complications following osteoporotic fractures. Specifically, compared to patients aged 50–59 years, those in the 70–79 age group demonstrated a significantly higher risk of developing somatic complications [28]. Additionally, constipation represents one of the most prevalent gastrointestinal dysfunctions in middle-aged and elderly populations, with its incidence increasing progressively with age. Age-related neuronal loss mediates alterations in colonic motility and rectal sensitivity, collectively contributing to functional defecation disorders in this demographic [29,30]. Furthermore, advancing age is associated with significant changes in body composition and energy reserves, predisposing individuals to hypoproteinemia and malnutrition through multiple pathological mechanisms [31–33]. Recent studies have identified the Prognostic Nutritional Index (PNI) as a predictive factor for postoperative complications and 2-year mortality in hip fracture patients [34], highlighting the critical role of nutritional status in determining clinical outcomes. The fracture-induced trauma triggers an inflammatory response that reduces serum albumin levels, leading to hypoalbuminemia. Notably, research has demonstrated that low serum albumin serves as an indicator of inflammatory severity [35]. This inflammatory cascade increases intestinal capillary permeability, causing mucosal edema and subsequent malabsorption of nutrients [36]. Extensive evidence confirms that chronic malnutrition is associated with persistently low albumin levels, which has been widely adopted as a biomarker of nutritional status [37]. When patients experience prolonged malnutrition, it results in gastric mucosal atrophy, decreased neuromuscular cellularity, and impaired intestinal motility, collectively exacerbating constipation development. A prospective cohort study [38] demonstrated that dietary dairy supplementation significantly improved self-reported gastrointestinal symptoms (including constipation and bloating) through gut microbiota modulation, suggesting that nutritional intervention represents an effective strategy for constipation prevention. Malnutrition, a well-established risk factor for functional constipation in the elderly populations, has been shown to concurrently increases the incidence of other postoperative adverse events [33,39]. For instance, it can prolong the length of hospital stay, with studies demonstrating an approximately 50% prevalence among inpatients-particularly middle-aged and elderly patients. This risk exhibits a clear duration-dependent relationship, where longer hospital stays correlate with both higher constipation incidence and greater the impact on patients’ quality of life [40]. Notably, constipation is particularly prevalent among middle-aged and elderly in-patients with lower limb fractures, especially those with femoral fractures. It not only affects the rehabilitation efficiency of patients but also reduces the functional recovery of the body after discharge [41–43]. Multiple studies have also indicated that the fracture site directly influences the occurrence of postoperative complications. For example, pulmonary complications are common after hip fracture surgery [44], while infections are more prevalent after ankle fracture surgery [45,46]. Although constipation is a common postoperative complication among middle-aged and elderly patients with lower extremity fractures, its incidence varies significantly depending on fracture location due to differential impacts on mobility duration. Notably, we identified chronic gastritis as an independent risk factor for constipation, consistent with the findings reported by Liu [14]et al. A multicenter Chinese study [47] demonstrated that chronic gastritis—characterized by gastric mucosal inflammation primarily caused by Helicobacter pylori infection—is highly prevalent, with a mean patient age of approximately 50 years. H. pylori infection disrupts gastric microbiota composition, reduces gastric acid secretion, and delays gastric emptying, ultimately impairing intestinal motility and contributing to bowel dysfunction [48]. These findings confirm the clinical and biological plausibility of the predictive factors identified by our algorithm, reinforcing its reliability.

This study demonstrates significant potential for clinical translation through three key applications: First, the predictive model can be integrated into electronic medical record (EMR) systems to enable automated, real-time assessment and risk stratification of postoperative constipation. This implementation would enhance clinical vigilance for high-risk patients by providing immediate access to critical indicators (e.g., age, malnutrition parameters) during admission evaluations. Second, the identified risk factors facilitate targeted interventions, including prophylactic bowel management for elderly patients and nutritional support for those at metabolic risk. Finally, this approach optimizes clinical decision-making and resource allocation, demonstrating dual benefits of reduced healthcare costs and improved patient outcomes-particularly when incorporated into Enhanced Recovery After Surgery (ERAS) protocols. The model’s reliance on routinely collected EMR data ensures immediate implementation feasibility while maintaining alignment with precision medicine initiatives.

## Conclusion

This study successfully developed and validated a predictive model for the risk of postoperative constipation in middle-aged and elderly patients with lower limb fractures based on machine learning algorithms, demonstrating that the logistic regression model achieved optimal performance in both predictive accuracy (AUC = 0.736) and clinical applicability. Through interpretability analysis, we identified several clinically significant predictors including advanced age, femoral fracture, prolonged hospital stay, nutritional risk, and chronic gastritis, all of which showed strong concordance with established clinical evidence and can facilitate early risk identification and preventive interventions by healthcare providers. This study has limitations as a single-center retrospective analysis without external validation, which precludes definitive conclusions about the model’s generalizability. Nevertheless, it provides valuable evidence-based insights for postoperative complication prevention. Future research directions include multicenter prospective validation to assess the model’s generalizability and development of an integrated risk monitoring system incorporating dynamic postoperative indicators, ultimately aiming to optimize comprehensive fracture management protocols.
